# Self-Reported Health Literacy and Current Conventional Cigarette Smoking Among Korean Adults: A Cross-Sectional Analysis of the 2024 Korea National Health and Nutrition Examination Survey

**DOI:** 10.3390/healthcare14172866

**Published:** 2026-09-06

**Authors:** Jiyoung Kim, Hyeokjae Kwon

**Affiliations:** 1 College of Nursing, Woosuk University, 443 Samnye-ro, Samnye-eup, Wanju 55338, Republic of Korea; 2 Department of Plastic and Reconstructive Surgery, College of Medicine, Chungnam National University, 266 Munhwa-ro, Jung-gu, Daejeon 35015, Republic of Korea; 3 Department of Plastic and Reconstructive Surgery, Chungnam National University Hospital, 282 Munhwa-ro, Jung-gu, Daejeon 35015, Republic of Korea; 4 Department of Radiology, Massachusetts General Hospital, Boston, MA 02114, USA

**Keywords:** health literacy, cigarette smoking, Korea National Health and Nutrition Examination Survey, sampling studies

## Abstract

**Highlights:**

**What are the main findings?**

- In a survey-weighted analytic sample of 5792 adults from the nationally representative 2024 KNHANES, each 5-point-lower score on the Health Literacy Index for the Community (HLIC) was associated with a 10.3% higher adjusted prevalence of current conventional cigarette smoking.
- After standardization, smoking prevalence was 3.34 percentage points higher among adults with HLIC scores below 30 than among those with scores of 30 or higher. Separate comparisons with former smokers and adults who had smoked fewer than 100 lifetime cigarettes produced estimates in the same direction. The prevalence-ratio scale interaction test provided no clear evidence that the association varied by sex.

**What are the implications of the main findings?**

- The modest association may be relevant when smoking-prevention and cessation services are designed and evaluated.
- Longitudinal studies are needed to clarify temporal ordering, and intervention studies should test whether adapting services to different HLIC levels improves service use or smoking outcomes.

**Abstract:**

**Background/Objectives:** Analyses of the 2023 Korea National Health and Nutrition Examination Survey described health-literacy distributions by smoking status but did not model current conventional cigarette smoking across continuous Health Literacy Index for the Community (HLIC) scores or report a standardized absolute difference. **Methods:** We analyzed 5792 adults aged ≥19 years. Survey-weighted quasi-Poisson models estimated prevalence ratios (PRs) per 5-point-lower HLIC score, adjusting for age, sex, education, and household income. Standardized prevalences were compared for scores below versus at or above 30. Exploratory analyses separated former smokers from adults with fewer than 100 lifetime cigarettes and tested interaction by sex. **Results:** The adjusted PR per 5-point-lower score was 1.103 (95% confidence interval (CI), 1.020–1.192). Standardized smoking prevalence was 18.08% below 30 and 14.74% at or above 30; the difference was 3.34 percentage points (95% CI, 1.08–5.60). There was no clear evidence of sex-related heterogeneity on the PR scale (interaction *p* = 0.541), although the estimate among women was imprecise. **Conclusions:** Lower self-reported HLIC was associated with modestly higher smoking prevalence. The cross-sectional design precludes temporal inference, but the association may be relevant to the design and evaluation of accessible smoking-prevention and cessation services. Longitudinal and intervention studies are needed.

## 1. Introduction

Health literacy concerns how people find, understand, appraise, and use health information and services [1,2,3]. Population surveys measure this broad construct in different ways. Performance tests, knowledge questions, and self-report instruments are related but not interchangeable, and their results depend on the setting and purpose of measurement [4]. Recent scholarship has further emphasized that health literacy reflects not only individual capacities but also the demands imposed by health-information and service environments [5].

Health literacy may be relevant to smoking because interpreting tobacco warnings, comparing cessation options, discussing treatment with healthcare professionals, and navigating cessation services require people to access, understand, appraise, and apply information from multiple sources [6]. Meta-analytic evidence has linked lower health literacy with smoking and other health behaviors, although the underlying studies are heterogeneous and predominantly cross-sectional [7,8]. Among people who smoke, lower health literacy has also been associated with barriers to successful cessation [9]. The temporal ordering of HLIC and smoking remains uncertain, and both may reflect shared social conditions. Estimating their association therefore requires attention to major demographic and socioeconomic factors.

The Korea National Health and Nutrition Examination Survey (KNHANES) began administering the 10-item Health Literacy Index for the Community (HLIC) in 2023 [10]. Earlier 2023 KNHANES analyses described HLIC distributions by smoking status and examined correlates of HLIC within groups defined by smoking status and sex [11,12]. They did not model current conventional cigarette smoking across the continuous HLIC score, report a survey-standardized absolute prevalence difference, or assess the influence of smoking-item overlap and complete-case selection.

We therefore used the nationally representative 2024 KNHANES to estimate the association between continuous self-reported HLIC and current conventional cigarette smoking as both a prevalence ratio and a survey-standardized absolute contrast. We examined nonlinearity and robustness to alternative score construction and complete-case selection. Exploratory analyses separated former smokers from adults with fewer than 100 lifetime cigarettes and assessed variation by sex. We hypothesized that lower HLIC would be associated with a higher prevalence of current conventional cigarette smoking after adjustment for major demographic and socioeconomic factors.

## 2. Materials and Methods

### 2.1. Study Design and Participants

KNHANES is an annual, nationally representative survey conducted by the Korea Disease Control and Prevention Agency (KDCA) [13]. It uses a two-stage stratified cluster sample of the noninstitutionalized Korean population, with 192 primary sampling units selected in 2024 and 25 households sampled within each unit. We used the 2024 health interview and examination file and included adults aged 19 years or older. Characteristics of adults included in and excluded from the complete-case analysis are summarized in Supplementary Table S1.

We specified the survey design before restricting the data to adults or complete cases. All analyses incorporated the KNHANES stratification, clustering, and combined health interview-examination weights. The nutrition weight was not used because the analysis included no nutrition-survey variables.

### 2.2. Self-Reported Health Literacy

The HLIC is a validated 10-item self-report measure of respondents’ perceived ability to find, understand, appraise, and use health information; it is not a performance test [10]. The items cover disease prevention, health promotion, health care, and resource utilization. Responses are scored from 1 to 4, yielding a total of 10 to 40, with higher scores indicating greater perceived ability. Under the KNHANES scoring rule, an item coded as unknown or nonresponse contributes 1 point to the released total. We therefore distinguished the official released total from a score restricted to substantive responses on all 10 items. The official and alternative score constructions are summarized in Supplementary Table S2.

The primary exposure contrast was a 5-point-lower HLIC score, chosen for interpretability rather than from the score distribution of the analytic sample. As a secondary analysis, we reported associations per 1 survey-weighted standard deviation decrease. Scores below 30 and below 28 were used as secondary categorical definitions. The former is used in national surveillance and the latter arose during instrument development; neither is a clinical cutoff [10,11].

Item 3 asks whether respondents know the warning signs of health problems that may result from smoking, excessive drinking, or insufficient physical activity. Because this item overlaps explicitly with the smoking outcome, we recalculated the HLIC score after removing it. The resulting 9-item score was analyzed per 5 raw-score points and per its analysis-specific standard deviation.

### 2.3. Current Conventional Cigarette Smoking

The primary outcome was current conventional cigarette smoking, assessed in the self-administered health-behavior questionnaire. Current smokers were adults who had smoked at least 100 conventional cigarettes during their lifetime and reported smoking every day or occasionally at the time of the survey. Former smokers and adults who had smoked fewer than 100 cigarettes were classified as not currently smoking in the official binary surveillance outcome. To determine whether this heterogeneous comparison group obscured distinct patterns, exploratory analyses compared current established smokers separately with former established smokers and with adults who had smoked fewer than 100 lifetime cigarettes. These cross-sectional contrasts were not interpreted as effects on smoking initiation or cessation.

The primary outcome was restricted to conventional cigarettes to retain comparability with the official KNHANES surveillance indicator. Electronic cigarettes and heated tobacco products were not incorporated because their current-use questions differ from the conventional-cigarette indicator, and KNHANES does not provide an official composite outcome across these products. Consequently, exclusive users of nonconventional products could be classified as noncurrent conventional-cigarette smokers.

### 2.4. Covariates

Covariates were specified a priori. Age and sex were included as demographic factors, with age modeled using a natural cubic spline with 3 degrees of freedom. Education and household income represented socioeconomic conditions potentially related to both HLIC and smoking. Occupation was not included because it did not apply uniformly to all adults and overlapped substantially with education and income. Mental health, healthcare use, and nicotine-dependence variables were not treated as baseline confounders because their temporal relationships with HLIC and smoking were uncertain, and some were available only for people who smoked.

We fitted an unadjusted model, a model adjusted for age and sex, and the primary adjusted model, which additionally included education and household income. The primary estimate was interpreted as an association conditional on these measured covariates; residual confounding remained possible.

### 2.5. Statistical Analysis

Participant characteristics were summarized using survey-weighted means or percentages and design-based 95% confidence intervals (CIs). We did not use significance tests to compare HLIC categories.

Survey-weighted quasi-Poisson regression with a log link was used to estimate prevalence ratios. For a binary outcome, the quasi-Poisson specification yields the same coefficients as the corresponding Poisson log-link model while relaxing the Poisson mean–variance assumption. Taylor-linearized design-based sandwich variances accounted for stratification and clustering, and inference used the survey design degrees of freedom with t or F reference distributions, as appropriate. This approach estimates prevalence ratios directly while avoiding convergence problems that may arise with log-binomial models. The primary design contained 27 strata, 192 primary sampling units (PSUs), and 165 design degrees of freedom; each stratum contained at least two PSUs.

Departure from linearity was assessed by a design-based Wald comparison of models containing a linear HLIC term or a natural spline with 3 degrees of freedom; the 5-point PR was interpreted as an average log-linear summary. Standardized prevalence curves were estimated from a survey-weighted quasi-binomial spline model with a logit link and standardized to the weighted analytic sample. Delta-method confidence intervals used the model covariance matrix.

Standardized prevalences for HLIC scores below 30 and at or above 30 were estimated using weighted quasi-binomial regression with a logit link. The model and marginal predictions were recomputed in the full sample and each delete-one-PSU jackknife (JKn) replicate. JKn standard errors and 95% CIs used the survey design degrees of freedom. The standardized prevalence difference was treated as an adjusted descriptive contrast, not as a preventable fraction or intervention effect.

The primary analysis included participants with data for the released HLIC total, smoking, age, sex, education, and household income. We tabulated variable-specific and overlapping missingness and compared included and excluded participants using information available in both groups. Released totals of 10 were cross-classified with substantive item responses and unknown or nonresponse codes.

Complete-case selection was examined by modeling analysis inclusion with age and HLIC splines, sex, and any code-9 HLIC response. The inverse estimated inclusion probability was combined with the survey weight. Selection factors were trimmed above the 99th percentile in the primary sensitivity analysis and left untrimmed in a second analysis after predicted probabilities were bounded at 0.001 and 0.999. We examined effective sample size and the factor distribution; variance estimation treated selection factors as fixed.

Other sensitivity analyses used the 9-item score, restricted the sample to substantive responses on all HLIC items, excluded participants with the minimum released HLIC total of 10, and rescaled HLIC by the analysis-specific standard deviation.

In exploratory analyses, we separately compared current established smokers with adults who had smoked fewer than 100 lifetime cigarettes and with former established smokers. We also fitted an HLIC-by-sex interaction and obtained sex-specific PRs. For descriptive absolute contrasts, standardized probabilities for HLIC scores below 30 versus at or above 30 were estimated using weighted logistic models with JKn variance estimation. Status-pair models adjusted for age (natural spline, 3 df), sex, education, and household income; sex-specific models omitted sex. The sex-specific absolute contrasts were descriptive; no formal additive-scale interaction test or multiplicity adjustment was applied.

Analyses were conducted using R (version 4.6.1; R Foundation for Statistical Computing, Vienna, Austria) with the survey package (version 4.5). All tests were two-sided, with *p* < 0.05 denoting statistical significance. Model convergence and fitted values were examined; fitted probabilities from the primary quasi-Poisson model ranged from 0.005 to 0.897.

### 2.6. Ethics

The 2024 KNHANES protocol was approved by the Institutional Review Board of the Korea Disease Control and Prevention Agency (2022-11-16-R-03), and all participants provided written informed consent. This study was a secondary analysis of publicly available, de-identified data.

## 3. Results

### 3.1. Participant Flow and Characteristics

**Figure 1:**
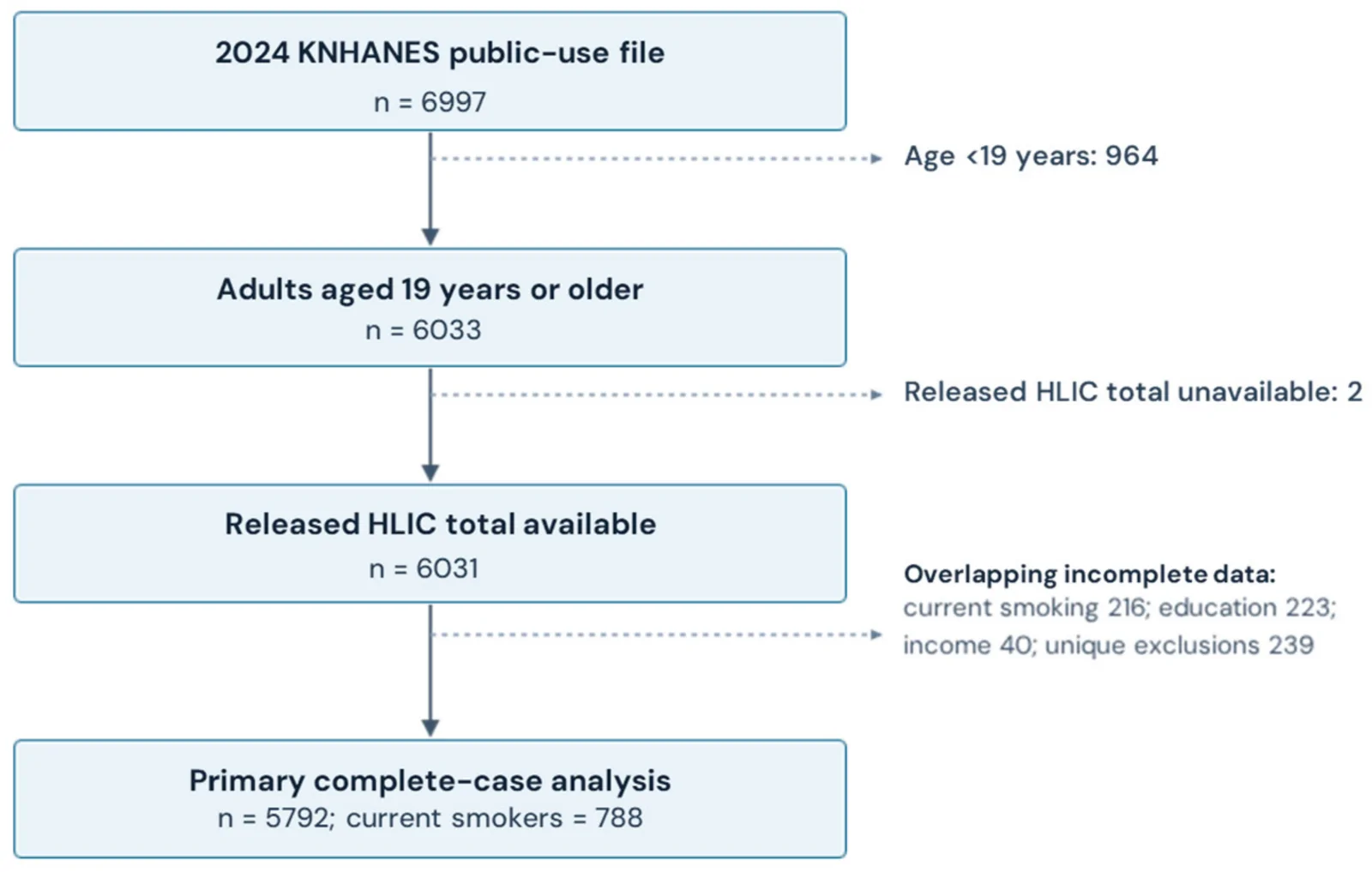
Flow of participants into the 2024 primary analysis. Counts are unweighted, and reasons for incomplete data overlap. HLIC, Health Literacy Index for the Community.

The 2024 public-use file contained 6997 participants, of whom 6033 were adults. A released HLIC total was available for 6031 adults, and 5792 had complete data for the primary model (Figure 1). Among adults with a released HLIC total, 216 lacked current-smoking information, 223 lacked education, and 40 lacked income. Missing smoking and education data overlapped in 216 adults; 24 of these also lacked income.

Inclusion in the complete-case analysis varied markedly with the released HLIC total. Included participants had a weighted mean HLIC score of 30.68, compared with 12.14 among the 239 excluded participants. Of those excluded, 90.5% had at least one unknown or nonresponse HLIC item and 90.3% had a released total of 10. Among the 258 adults with a released total of 10, 225 had at least one code-9 response, 222 had code 9 recorded for every item, and 33 had provided a response of 1 to all 10 items. Thus, a released total of 10 usually reflected the coding of unknown or nonresponse items as 1 rather than 10 observed responses of 1 (Supplementary Table S1).

In the analytic sample, the weighted mean HLIC score was 30.68 (survey-weighted standard deviation [SD], 4.89), and the weighted prevalence of current conventional cigarette smoking was 15.90% (95% CI, 14.57–17.22%). The sample included 3650 adults with HLIC scores of 30 or higher and 2142 with scores below 30. Adults with scores below 30 were older and had lower education and household income than those with higher scores (Table 1).

### 3.2. Primary Association and Absolute Prevalence Contrast

Per 5-point-lower HLIC score, the PR was 1.104 (95% CI, 1.028–1.185) before adjustment, 1.171 (95% CI, 1.084–1.264) after adjustment for age and sex, and 1.103 (95% CI, 1.020–1.192; *p* = 0.014) after further adjustment for education and household income (Table 2, Panel A). The corresponding primary adjusted PR per analysis-specific standard deviation was 1.101 (95% CI, 1.020–1.188).

The adjusted PR was 1.222 (95% CI, 1.069–1.396) for HLIC below 30 versus at or above 30 and 1.248 (95% CI, 1.054–1.477) for HLIC below 28 versus at or above 28. Standardized smoking prevalence was 18.08% (95% CI, 16.06–20.10%) below 30 and 14.74% (95% CI, 13.23–16.24%) at or above 30. The standardized prevalence difference was 3.34 percentage points (JKn 95% CI, 1.08–5.60; Table 2, Panel B).

**Figure 2:**
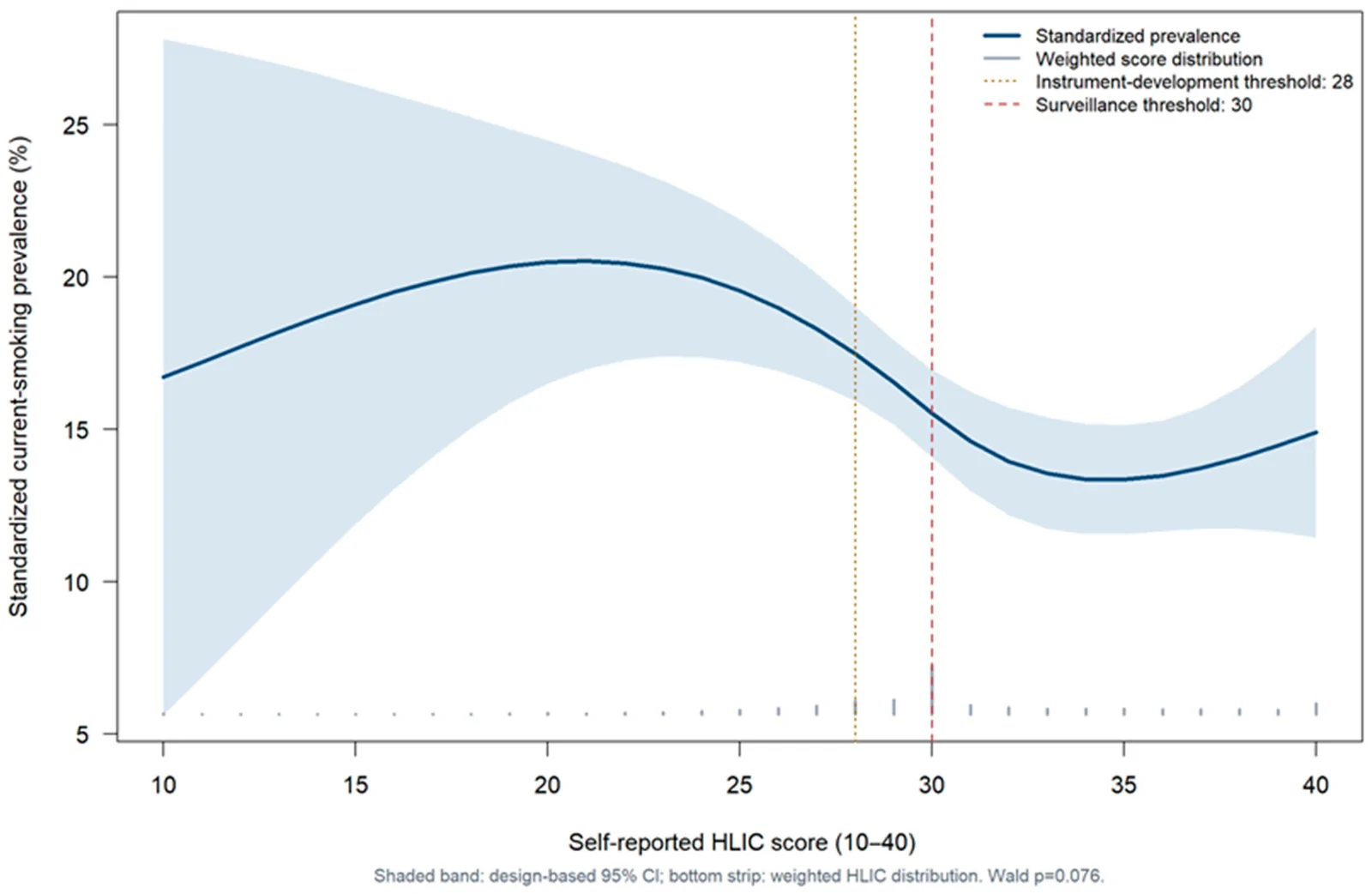
Standardized current conventional cigarette smoking prevalence across the self-reported HLIC score. The curve is adjusted for age, sex, education, and household income. Confidence intervals widen at sparsely observed score extremes. HLIC, Health Literacy Index for the Community.

The formal test did not provide strong evidence of departure from linearity (*p* = 0.076). The spline nevertheless showed some local curvature, although estimates at sparsely observed score extremes were imprecise (Figure 2).

### 3.3. Sensitivity Analyses

After item 3 was removed, the adjusted PR per 5-point-lower 9-item score was 1.110 (95% CI, 1.017–1.212); the corresponding estimate per analysis-specific standard deviation was 1.096 (95% CI, 1.015–1.184).

The PR per 5-point-lower HLIC was 1.100 (95% CI, 1.017–1.189) after excluding participants with at least one code-9 HLIC response and 1.104 (95% CI, 1.017–1.198) after excluding released totals of 10.

With inverse selection factors trimmed at the 99th percentile, the PR was 1.103 (95% CI, 1.020–1.192), the combined-weight effective sample size was 4557, and the factor cap was 1.031. In the untrimmed analysis, the PR was 1.138 (95% CI, 1.038–1.248); among complete cases, the minimum modeled inclusion probability was 0.054, the maximum inverse selection factor was 18.465, and the effective sample size was 4279. The untrimmed estimate was larger than the trimmed and complete-case estimates and was more sensitive to the specified inclusion model. Primary and sensitivity estimates are summarized in Table 3 and displayed in Supplementary Figure S1.

The survey-weighted internal-consistency coefficient for the 10 HLIC items was 0.919, and the weighted correlation between the 10- and 9-item scores was 0.995. Floor and ceiling percentages were 0.46% and 6.50%, respectively; 0.29% of the primary sample had at least one code-9 response (Supplementary Table S2).

### 3.4. Exploratory Analyses

When current established smokers were compared separately with adults who had smoked fewer than 100 lifetime cigarettes and with former established smokers, the adjusted PRs per 5-point-lower HLIC score were 1.073 (95% CI, 1.005–1.145) and 1.074 (95% CI, 1.000–1.152), respectively (Supplementary Table S3). Estimates were similar in direction and magnitude in both comparisons. Because HLIC and smoking status were measured concurrently, the comparison with former smokers does not provide evidence about cessation.

The adjusted PR per 5-point-lower HLIC score was 1.111 (95% CI, 1.018–1.213) among men and 1.051 (95% CI, 0.903–1.224) among women. The women-to-men ratio of PRs was 0.946 (95% CI, 0.791–1.131; interaction *p* = 0.541) (Supplementary Table S3). The estimate among women was imprecise, with 126 current smokers.

## 4. Discussion

In the nationally representative 2024 KNHANES, lower self-reported HLIC was associated with a modestly higher prevalence of current conventional cigarette smoking after adjustment for age, sex, education, and household income. The adjusted prevalence ratio and standardized prevalence difference pointed in the same direction, and the estimate changed little in most sensitivity analyses. These cross-sectional estimates should be interpreted as associations; they do not show that lower HLIC causes smoking or that increasing HLIC would reduce it.

The finding was consistent with our prespecified hypothesis and with previous Korean evidence. In the 2023 KNHANES, the proportion classified as having adequate HLIC was lower among current smokers than among nonsmokers, but that analysis described HLIC distributions by smoking status rather than modeling current smoking prevalence across the continuous score [11]. In the 2021 Korea Community Health Survey, difficulty understanding spoken or written health information was associated with current cigarette smoking after covariate adjustment, although those items represented a narrower aspect of health literacy than HLIC [6]. A meta-analysis likewise reported higher odds of smoking among adults with inadequate health literacy, while noting substantial heterogeneity across instruments and study populations [7]. More recently, a survey of men in Ningbo City found an inverse association between health literacy and smoking [14]. Differences in measurement, sex composition, study populations, and effect measures preclude direct comparison of effect sizes. The present PR of 1.103 therefore supports a consistent direction of association while indicating a modest magnitude in Korean adults. The present study extends earlier Korean evidence by modeling current smoking prevalence across the continuous HLIC score, reporting both prevalence ratios and standardized prevalence differences, and examining robustness to the smoking-related item, released-score coding, and complete-case selection [11,12].

As a self-report measure, HLIC may capture confidence, prior healthcare experience, access to digital resources, and response style alongside perceived ability to understand health information. Several pathways could account for the observed association. Lower health literacy has been associated with less knowledge of smoking harms, lower risk perception and self-efficacy, more favorable smoking expectancies, and greater nicotine dependence [8,9,15]. These factors may influence how people interpret tobacco warnings or use cessation services. However, HLIC and smoking may also reflect shared socioeconomic conditions, and the present data cannot establish mediation or temporal ordering. These pathways should therefore be regarded as hypotheses for longitudinal and intervention studies. The estimate changed little after the smoking-related item was removed, indicating that the association was not dependent on that item alone. Overlap with other health-promotion items and bias from shared self-report nevertheless remain possible.

Complete-case inclusion was closely related to the released HLIC total. Because the scoring rule assigns the lowest item score to unknown or nonresponse codes, the very low totals observed among many excluded adults did not necessarily represent low measured ability. The trimmed selection-weighted estimate was close to the primary estimate, whereas the larger untrimmed estimate showed sensitivity to the specified inclusion model; neither approach eliminates uncertainty about selection bias.

Restricting the outcome to conventional cigarettes retained comparability with the official KNHANES indicator but excluded adults who used heated tobacco or vaping products without conventional cigarettes. This distinction is important in Korea, where concurrent use of conventional cigarettes, heated tobacco products, and nicotine-vaping products has been documented [16]. The findings therefore apply to current conventional cigarette smoking, not to tobacco or nicotine use as a whole.

The 3.34-percentage-point standardized difference had a confidence interval that excluded zero, but its absolute magnitude was modest. Because cross-sectional standardization balances measured covariates rather than simulating an intervention, this estimate should not be interpreted as the reduction in smoking expected from raising HLIC. Its practical importance will depend on the population reached. The association raises the question of whether smoking information, counseling, and referral pathways are equally understandable and easy to navigate across HLIC levels; it does not show that redesigning these services would change smoking behavior.

Use of a national probability sample and design-based analysis supports population-level interpretation. The comparable estimates in comparisons with adults who had smoked fewer than 100 lifetime cigarettes and with former established smokers suggest that the main association was not driven by combining two noncurrent groups with opposing patterns. However, the current-versus-former comparison is cross-sectional and does not estimate cessation. We found no clear evidence of sex-related heterogeneity on the prevalence-ratio scale. Earlier Korean reports described sex differences in HLIC distributions or correlates [11,12], whereas our interaction analysis addressed whether the HLIC–smoking association varied by sex. This is not evidence of equivalence. The estimate among women was imprecise because only 126 women were classified as current smokers. Separately, underreporting of self-reported smoking among Korean women may have biased the sex-specific estimate. Additive-scale interaction was not formally tested, and the sex-specific standardized differences were interpreted descriptively.

The overall Wald test did not provide strong evidence against linearity; nevertheless, it does not exclude localized departures, and sparse data at the score extremes preclude confident characterization of those regions. The per-5-point PR should therefore be interpreted as an average log-linear association across the observed score distribution.

Prospective studies should assess whether adapting smoking information, counseling, and referral pathways to different HLIC levels improves their reach, uptake, and subsequent smoking outcomes. Longitudinal analyses should distinguish initiation, continued smoking, and cessation and include heated tobacco and vaping products where possible.

### Limitations

Because HLIC and smoking were measured concurrently, their temporal order could not be determined. Residual confounding remains possible despite adjustment for major demographic and socioeconomic factors; occupation, material hardship, mental health, nicotine dependence, healthcare contact, and social context were incompletely measured or temporally ambiguous. The sampling frame excluded institutionalized adults, and residual bias from survey nonresponse cannot be ruled out despite the use of survey weights.

Both HLIC and conventional cigarette smoking were self-reported. The HLIC has been validated in previous work and showed high internal consistency in this sample, but the smoking responses were not verified against cotinine in the present study. Social-desirability bias and underreporting may therefore remain, particularly among Korean women [17]. Although exploratory analyses separated former smokers from adults with fewer than 100 lifetime cigarettes, the survey cannot determine whether differences in HLIC preceded smoking initiation, accompanied continued smoking, or changed after cessation. The outcome also did not capture quit attempts, intensity, duration, or exclusive use of nonconventional products.

Complete-case restriction disproportionately removed adults with very low released HLIC totals, creating potential selection bias. The estimate was close to the primary result after trimming inverse selection factors at the 99th percentile but larger without trimming. This sensitivity analysis depends on the specified inclusion model, assumes a nonzero probability of inclusion, and treats the estimated factors as fixed; it therefore characterizes, but cannot remove, uncertainty arising from complete-case selection.

Spline estimates were imprecise at sparsely observed HLIC scores, and the average linear PR may conceal local departures. The thresholds of 28 and 30 arose from instrument development and national surveillance, respectively, and are not diagnostic cutoffs. Finally, the status- and sex-specific analyses were exploratory, and no adjustment for multiple comparisons was applied.

## 5. Conclusions

In the 2024 KNHANES, lower self-reported HLIC was associated with a modestly higher prevalence of current conventional cigarette smoking after adjustment for age, sex, education, and household income. Because HLIC and smoking were measured concurrently, the temporal order of this relationship remains uncertain. Even so, the findings may be relevant to the design and evaluation of smoking-prevention and cessation services that are easy to understand and navigate. Longitudinal studies should distinguish initiation, continued smoking, and cessation, while intervention studies should determine whether adapting services to different HLIC levels improves service use or smoking outcomes.

## Figures and Tables

**Table 1:** Characteristics of the 2024 primary analytic sample.

Characteristic	Overall (*n* = 5792)	HLIC ≥ 30 (*n* = 3650)	HLIC < 30 (*n* = 2142)
Age, years	49.6 (48.7–50.6)	47.3 (46.5–48.2)	54.0 (52.7–55.4)
Sex: men	50.1 (48.9–51.3)	49.5 (48.0–51.0)	51.3 (49.3–53.4)
women	49.9 (48.7–51.1)	50.5 (49.0–52.0)	48.7 (46.6–50.7)
Education: elementary or less	11.5 (10.0–12.9)	5.8 (4.8–6.8)	22.1 (19.6–24.7)
middle school	8.0 (7.1–8.8)	5.6 (4.8–6.4)	12.4 (10.8–13.9)
high school	34.3 (32.4–36.2)	33.9 (31.7–36.1)	35.1 (32.2–37.9)
college or higher	46.3 (43.6–49.0)	54.6 (51.8–57.5)	30.4 (27.3–33.6)
Household income: low	13.9 (12.2–15.6)	10.1 (8.6–11.6)	21.1 (18.4–23.9)
lower-middle	22.8 (20.9–24.8)	22.1 (20.0–24.3)	24.1 (21.5–26.7)
upper-middle	29.9 (27.7–32.2)	30.5 (28.0–33.1)	28.8 (25.8–31.7)
high	33.3 (30.1–36.5)	37.2 (33.6–40.8)	25.9 (22.7–29.2)
Current conventional smoking: no	84.1 (82.8–85.4)	85.5 (84.0–87.0)	81.5 (79.3–83.7)
yes	15.9 (14.6–17.2)	14.5 (13.0–16.0)	18.5 (16.3–20.7)

Values are survey-weighted mean or percentage (95% CI); *n* values are unweighted. No between-group hypothesis tests were performed. HLIC, Health Literacy Index for the Community; CI, confidence interval.

**Table 2:** Association between self-reported HLIC and current conventional cigarette smoking, 2024 KNHANES.

**Panel A. Prevalence ratios for current conventional cigarette smoking according to HLIC score.**							
**Model**	***n***		**PR**		**95% CI**		***p***
Unadjusted	5792		1.104		1.028–1.185		0.007
Age and sex	5792		1.171		1.084–1.264		<0.001
Age, sex, education, and income	5792		1.103		1.020–1.192		0.014
Secondary scaling: 1 analytic-sample SD lower (4.893 points)	5792		1.101		1.020–1.188		0.014
**Panel B. Standardized smoking prevalence and prevalence difference according to HLIC category.**							
**Estimand**		**Estimate**		**95% CI**		**Method**	
HLIC ≥ 30 prevalence		14.74%		13.23–16.24		Delete-one-PSU JKn	
HLIC < 30 prevalence		18.08%		16.06–20.10		Delete-one-PSU JKn	
Prevalence difference		3.34 pp		1.08–5.60		Delete-one-PSU JKn	

Primary PRs are per 5-point-lower HLIC score. The full model includes age (natural spline, 3 df), sex, education, and household income. The analytic design contained 27 strata, 192 PSUs, and 165 design degrees of freedom. HLIC, Health Literacy Index for the Community; PR, prevalence ratio; df, degrees of freedom; PSU, primary sampling unit. CI, confidence interval; JKn, jackknife with one primary sampling unit deleted at a time; pp, percentage points.

**Table 3:** Sensitivity analyses for self-reported HLIC and current conventional cigarette smoking, 2024 KNHANES.

Analysis	Score or Sample Definition	Adjusted PR	95% CI
Primary analysis	Released 10-item HLIC total	1.103	1.020–1.192
Item 3 removed	9-item HLIC score	1.110	1.017–1.212
Substantive responses on all items	Released 10-item HLIC total	1.100	1.017–1.189
Minimum released HLIC total (10) excluded	Released 10-item HLIC total	1.104	1.017–1.198
Selection-IPW, 99th-percentile trimmed	Released 10-item HLIC total	1.103	1.020–1.192
Selection-IPW, untrimmed	Released 10-item HLIC total	1.138	1.038–1.248

All estimates are adjusted for age, sex, education, and household income and are expressed per 5-point-lower score. Selection-IPW estimates combine the survey weight with the inverse estimated probability of analysis inclusion; estimated selection factors were treated as fixed for variance estimation. CI, confidence interval; HLIC, Health Literacy Index for the Community; IPW, inverse probability weighting; PR, prevalence ratio.

## Data Availability

The public-use KNHANES data are available from the Korea Disease Control and Prevention Agency KNHANES website (https://knhanes.kdca.go.kr (accessed on 2 July 2026)) after completion of the applicable data-use procedures. Additional analytic materials generated for this study are available from the corresponding author upon reasonable request, subject to KDCA data-use conditions.

## Supplementary Materials

The following supporting information can be downloaded at: https://www.mdpi.com/article/10.3390/healthcare14172866/s1, Table S1: Included versus excluded HLIC-available adults, 2024 KNHANES; Table S2: HLIC score construction and measurement characteristics, 2024 KNHANES; Table S3: Exploratory smoking-status and sex-specific analyses; Figure S1: Primary and sensitivity estimates.
