# Supplementary material for: Self-Reported Health Literacy and Current Conventional Cigarette Smoking Among Korean Adults: A Cross-Sectional Analysis of the 2024 Korea National Health and Nutrition Examination Survey
Source: Healthcare (Basel). 2026 Sep 6;14(17):2866. doi: 10.3390/healthcare14172866 (PMC13565434; doi:10.3390/healthcare14172866)
Supplement: Supplementary file 1 [file healthcare-14-02866-s001.zip › healthcare-4513765-supplementary.pdf]

Supplementary Materials: Self-Reported Health Literacy and Current Conventional Cigarette Smoking Among Korean Adults: A Cross-Sectional Analysis of the 2024 Korea National Health and Nutrition Examination Survey

Jiyoung Kim [1] and Hyeokjae Kwon [2,3,4,*]

[1] College of Nursing, Woosuk University, 443 Samnye-ro, Samnye-eup, Wanju 55338, Republic of Korea
[2] Department of Plastic and Reconstructive Surgery, College of Medicine, Chungnam National University, 266 Munhwa-ro, Jung-gu, Daejeon 35015, Republic of Korea
[3] Department of Plastic and Reconstructive Surgery, Chungnam National University Hospital, 282 Munhwa-ro, Jung-gu, Daejeon 35015, Republic of Korea
[4] Department of Radiology, Massachusetts General Hospital, Boston, MA 02114, USA
* Correspondence: kwon.hyeokjae@cnuh.co.kr

Supplementary Materials

Supplementary Table S1. Included versus excluded HLIC-available adults, 2024 KNHANES.

Group	n	HLIC mean (SD)	Age	Men, %	Score 10, %	Any code 9, %
Included	5,792	30.68 (4.89)	49.6	50.1	0.5	0.3
Excluded	239	12.14 (6.79)	57.8	42.8	90.3	90.5

Percentages and means are survey-weighted; n is unweighted. Code 9 denotes unknown/nonresponse scored as 1 in the released total. HLIC, Health Literacy Index for the Community; SD, standard deviation.

Supplementary Table S2. HLIC score construction and measurement characteristics, 2024 KNHANES.

Panel A. Score construction used in the primary and sensitivity analyses.

Score	Construction	Range	Role
Official 10-item released total	Ten released item scores; unknown/nonresponse code contributes 1 point	10–40	Primary
Substantive-response 10-item score	Ten items restricted to responses 1–4	10–40	Sensitivity
Nine-item score	Researcher-derived score using the released-item scoring convention after excluding smoking-related item 3	9–36	Sensitivity

Panel B. Measurement characteristics in the primary analytic sample.

Metric	Estimate
Weighted Cronbach alpha	0.919
10-item/9-item correlation	0.995
Floor (score 10)	0.46%
Ceiling (score 40)	6.50%
Any code 9	0.29%

HLIC, Health Literacy Index for the Community. The score of 30 is a national-surveillance threshold rather than a clinical cutoff. The 9-item score was analyzed per 5 raw-score points and per its analysis-specific standard deviation.

Supplementary Table S3. Exploratory smoking-status and sex-specific analyses.

Panel A. Comparisons according to conventional-cigarette smoking status.

Restricted comparison	n	Current, n	Adjusted PR (95% CI)	p	HLIC ≥30	HLIC <30	Difference, pp (95% CI)
Current established vs <100 lifetime cigarettes	4,547	788	1.073 (1.005–1.145)	0.035	19.37%	22.86%	3.49 (1.04–5.94)
Current established vs former established	2,033	788	1.074 (1.000–1.152)	0.050	38.82%	45.12%	6.30 (1.41–11.19)

Current established smokers had smoked at least 100 lifetime cigarettes and smoked daily or occasionally; former established smokers had smoked at least 100 lifetime cigarettes but did not currently smoke. Standardized probabilities refer to being a current established smoker rather than having the stated comparison status within each restricted pairwise sample; they are not population prevalences of current smoking. PRs are per 5-point lower HLIC score. Both PR and absolute models adjusted for age (natural spline, 3 df), sex, education, and household income; absolute contrasts used weighted logistic models with delete-one-PSU JKn variance. CI, confidence interval; HLIC, Health Literacy Index for the Community; JKn, jackknife with one primary sampling unit deleted at a time; pp, percentage points; PR, prevalence ratio.

Panel B. Sex-specific associations.

Sex	n	Current, n	Weighted prevalence	Adjusted PR (95% CI)	p	HLIC ≥ 30	HLIC < 30	Difference, pp (95% CI)
Men	2,491	662	27.95%	1.111 (1.018–1.213)	0.019	25.68%	32.12%	6.44 (2.25–10.62)
Women	3,301	126	3.79%	1.051 (0.903–1.224)	0.517	3.67%	4.03%	0.36 (−1.13 to 1.85)

Sex-specific PRs were obtained from the full-sample HLIC-by-sex model adjusted for age (natural spline, 3 df), education, and household income. The women-to-men ratio of PRs was 0.946 (95% CI, 0.791–1.131; interaction p = 0.541). Separate sex-specific weighted logistic models adjusted for age (natural spline, 3 df), education, and household income and used JKn variance for standardized prevalences and differences. These absolute contrasts were descriptive; no formal additive-scale interaction test was performed. The analyses were exploratory, and no adjustment for multiple comparisons was applied.

Supplementary Figure S1. Primary and sensitivity estimates.

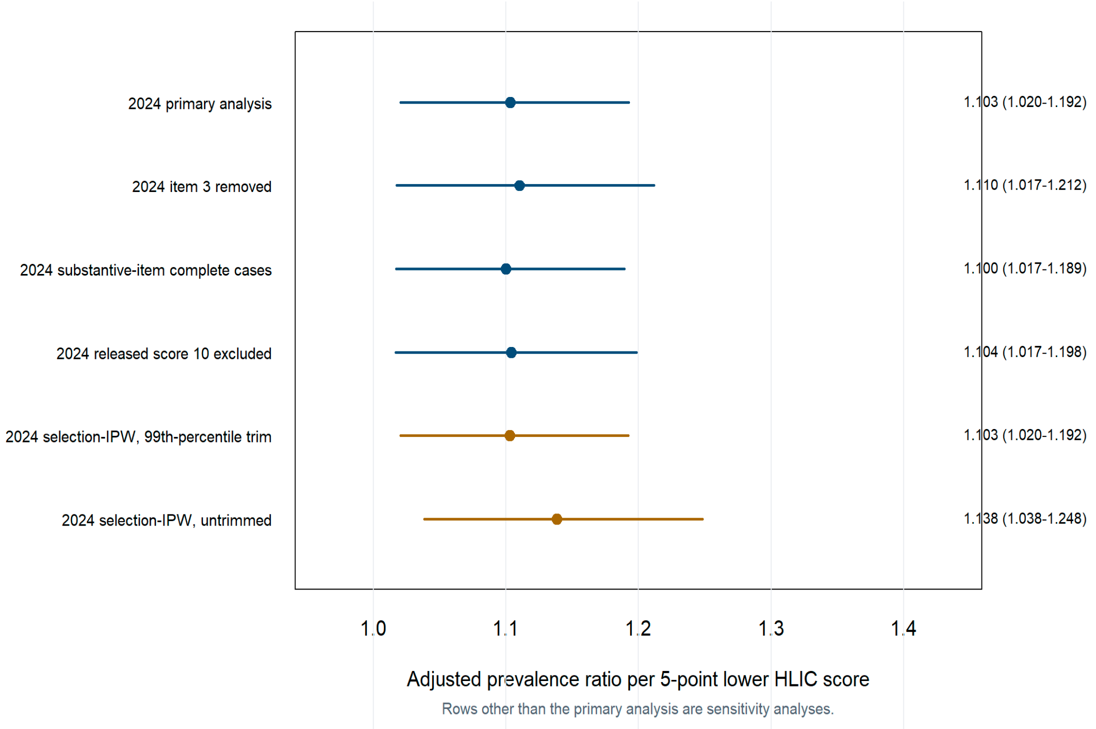

Points are adjusted PRs per 5-point lower HLIC score; horizontal lines are 95% CIs. CI, confidence interval; HLIC, Health Literacy Index for the Community; IPW, inverse probability weighting; PR, prevalence ratio.
